# Prediction of Responsiveness of Gait Variables to Rehabilitation Training in Parkinson's Disease

**DOI:** 10.3389/fneur.2019.00826

**Published:** 2019-08-02

**Authors:** Mariano Serrao, Giorgia Chini, Guido Caramanico, Michelangelo Bartolo, Stefano Filippo Castiglia, Alberto Ranavolo, Carmela Conte, Teresa Venditto, Gianluca Coppola, Cherubino di Lorenzo, Patrizio Cardinali, Francesco Pierelli

**Affiliations:** ^1^Department of Medical and Surgical Sciences and Biotechnologies, Sapienza University of Rome, Rome, Italy; ^2^Movement Analysis LAB, Policlinico Italia, Rome, Italy; ^3^Neurorehabilitation Unit, Department of Rehabilitation, Bergamo, Italy; ^4^Department of Occupational and Environmental Medicine, Epidemiology and Hygiene, INAIL, Rome, Italy; ^5^IRCCS – Fondazione Don Gnocchi, Milan, Italy; ^6^IRCCS – Fondazione Bietti, Rome, Italy; ^7^Unità Operativa Complessa Neurologia Area Vasta 4, Fermo, Italy; ^8^IRCCS - Neuromed, Pozzilli, Italy

**Keywords:** gait analysis, rehabilitation, Parkinson's disease, gait improvement, multiple linear regression

## Abstract

**Background:** Gait disorders represent one of the most disabling features of Parkinson's disease, which may benefit from rehabilitation. No consistent evidence exists about which gait biomechanical factors can be modified by rehabilitation and which clinical characteristic can predict rehabilitation-induced improvements.

**Objectives:** The aims of the study were as follows: (i) to recognize the gait parameters modifiable by a short-term rehabilitation program; (ii) to evaluate the gait parameters that can normalize after rehabilitation; and (iii) to identify clinical variables predicting improvements in gait function after rehabilitation.

**Methods:** Thirty-six patients affected by idiopathic Parkinson's disease in Hoehn-Yahr stage 1–3 and 22 healthy controls were included in the study. Both clinical and instrumental (gait analysis) evaluations were performed before and after a 10-weeks rehabilitation treatment. Time-distance parameters, lower limb joint, and trunk kinematics were measured.

**Results:** At baseline evaluation with matched speed, almost all gait parameters were significantly different between patients and healthy controls. After the 10-weeks rehabilitation, most gait parameters improved, and spatial asymmetry and trunk rotation normalized. Multiple linear regression of gender combined with Unified Parkinson's Disease Rating Scale-III predicted both ΔSpeed and ΔStep length of both sides; gender combined with Unified Parkinson's Disease Rating Scale-II predicted ΔCadence; age combined with Hoehn-Yahr score and disease duration predicted Δtrunk rotation range of motion.

**Conclusions:** Impaired gait parameters are susceptible to improvement by rehabilitation, and younger men with Parkinson's disease who are less severely affected and at early disease stage are more susceptible to improvements in gait function after a 10-weeks rehabilitation program.

## Introduction

Gait disorder represents one of the most disabling features of Parkinson's disease (1, 2) because it increases the risk of falls (3) and strongly affects patients' independence and quality of life (4, 5). The social and economic impact (6, 7), and the relationship of gait outcomes with longevity (8), cognitive decline (9), and other adverse events (10), necessitate gait rehabilitation to be considered as one of the primary focus of interventions in people with Parkinson's disease.

The mechanism underlying gait impairment is multi-factorial, reflecting global motor impairment. It is mainly related to neurotransmitter deficiency inducing bradykinesia, rigidity, abnormal trunk control, postural instability, visual motor impairment, and cognition (1, 11–13).

In addition to the characteristic shortened gait, recent studies have further detailed the gait pattern in people with Parkinson's disease using three-dimensional (3D) motion analysis, describing abnormalities in cadence, stance duration, swing duration, double support duration, step length, velocity, as well as ranges of motion (ROMs) of hip, knee, and ankle joints (14–18). These abnormal gait parameters seem to correlate with some clinical outcomes, such as the Unified Parkinson's Disease Rating Scale (UPDRS), the Hoehn and Yahr (H-Y) scale, as well as the dose of levodopa taken (14, 19). Importantly, several rehabilitation strategies, including physiotherapy, assistive equipment usage, sensory cueing, treadmill training, physical activity, and home-based exercises (20–22) can improve gait disorder and normalize gait parameters. However, none of the previous studies specifically investigated biomechanical factors affecting gait that can be modified after rehabilitation and the clinical characteristics that can predict rehabilitation-induced improvement. This information would be relevant to selecting, grouping, and typifying patients, thus optimizing rehabilitation strategies and cost management.

The aims of the present study were as follows: (i) to recognize the gait parameters that are modifiable by a short-term rehabilitation program; (ii) to evaluate the gait parameters that can normalize after rehabilitation and their responsiveness to rehabilitation treatment; and (iii) to identify clinical variables that can predict improvement of gait function after rehabilitation in a sample of people with Parkinson's disease. Specifically, it is hypothesized that in people with Parkinson's disease, gait function may improve after rehabilitation by modifications in gait parameters, joint kinematics and trunk motion, and that some gait parameters may be more responsive than others to rehabilitation. It is also hypothesized that some clinical characteristics may predispose patients to the gait improvement induced by rehabilitation and thus predict changes in gait variables.

## Materials and Methods

### Study Population

Sixty-seven patients affected by idiopathic Parkinson's disease who consecutively registered for outpatient rehabilitation between May 2014 and April 2017 were assessed. The inclusion criteria were as follows: a diagnosis of idiopathic Parkinson's disease according to the UK Brain Bank Diagnostic Criteria (23), H-Y stages 1–3, stable drug program (taking current medication for at least 2 weeks), and the ability to walk independently on at least the 8-meter long laboratory pathway in our facility without showing freezing of gait. The exclusion criteria were as follows: cognitive deficit [defined as scores of <24 on the Mini-Mental State Examination (MMSE)], moderate or severe depression [defined as scores of ≥20 on the Beck Depression Inventory (BDI)], and presence of orthopedic and/or other gait-influencing conditions such as arthrosis or total hip joint replacement.

Disease severity was evaluated using UPDRS-II and III and the H-Y staging system. For group comparison, 22 healthy subjects were included in the study, constituting a control group. This study was performed in accordance with the recommendations of Declaration of Helsinki with written informed consent from all subjects. The protocol was approved by the ethics committee “Sapienza University of Rome, Policlinico Umberto I” (protocol number UP 00263_2019). This study is registered as a clinical trial in ClinicalTrials.gov. (NCT03336307).

### Study Design

**Figure 1:**
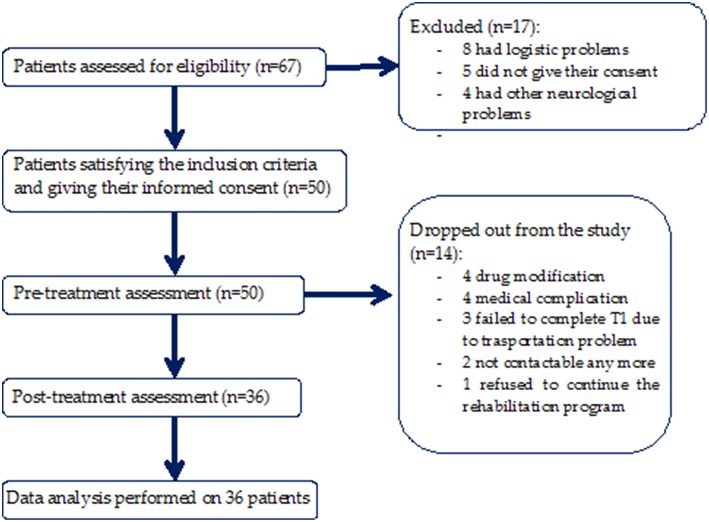
Study flow chart.

This was a bicentric observational study with a blind assessor (Figure 1). Both clinical and instrumental evaluations for gait analysis were performed at baseline before rehabilitation (T0) and after 10 weeks of rehabilitation treatment (T1). Treatment duration was in accordance with the duration of rehabilitation treatment of previous studies that demonstrated outcome changes (24–27).

Clinical evaluation included neurological and functional assessments using clinical scales. Medication was kept constant throughout the trial, and all interventions were performed at the same time of day for each patient during the “ON phase.” Participants were asked to maintain their usual activity levels and current medication dosage when not in the laboratory. Assessors for both clinical and instrumental evaluations were not involved in the treatment of the patients and were blinded to the time of the evaluation.

### Rehabilitation Program

All patients underwent rehabilitation treatment in accordance with the European Physiotherapy guidelines for PD (28).

The program focused on the following aspects: self-management support; prevention of inactivity and management of fear of falls; maintenance or improvement of global motor activities; improvement of physical performance; improvement of transfer abilities, balance, manual activities, and gait; reduction of pain; and delaying the onset of physical limitations.

Detailed exercises included the following:

- Standing up from and sitting down onto the floor
- Standing and walking on foam with and without perturbations (pushes and pulls) to the trunk
- Sitting down onto and rising from a chair while dual tasking
- Getting into and out of bed
- Rolling over in bed
- Walking with large steps and large amplitude arm swings
- Walking around and over obstacles
- Walking with sudden stops and changes in direction, including walking backwards
- Walking and maintaining balance while dual tasking, such as talking, carrying an object, or turning the head left to right to view wall mounted dots or photos and reporting what is seen
- Turning around in open, small, and narrow spaces
- Climbing steps.

The rehabilitation program comprised of one 60-min session/day, performed 3 days/week. Participants within the program were encouraged to progress based on pre-defined progression criteria by performing progression exercises including RoM exercises, stretching, upper and lower limb strengthening, and improving balance, standing, sitting, transferring, and walking.

### Instrumental Recordings

Gait analysis was performed by means of an optoelectronic motion analysis system (SMART-DX 500 System, BTS Engineering, Milan, Italy). The system consisted of eight infrared cameras, with a sampling frequency of 300 Hz, used to detect the movement of 22 passive spherical markers (15 mm in diameter) which were covered with a reflective aluminum powder and placed over prominent bony landmarks in accordance with a validated biomechanical model (29). Before the first data capture, a calibration procedure was performed. The spatial accuracy in three dimensions (x, y, z) was 0.2 mm, and a global reference system was adopted in accordance with the International Society of Biomechanics (30). Additionally, anthropometric measurements were obtained for each subject according to Winter's method (31), before the instrumental recordings.

### Experimental Procedure

Patients were asked to walk barefoot at a comfortable, self-selected speed along a walkway approximately 12 m in length while looking forward. Controls were requested to walk both at their preferred speed and at a lower speed. Since the interest of this study was natural locomotion, general qualitative instructions were provided. Before the recording session, all participants practiced for a few minutes to familiarize themselves with the procedure. At least six trials were recorded for each patient per session. To avoid muscle fatigue, each trial was separated by a 1-min rest period.

### Data Analysis

Three-dimensional (3D) marker trajectories were recorded using a frame-by-frame acquisition system (SMART Capture, BTS Engineering) and labeled using a frame-by-frame tracking system (SMART Tracker, BTS Engineering). Marker position data were interpolated, low-pass filtered using a zero-lag fourth-order Butterworth filter (6 Hz), and analyzed using 3D reconstruction software (SMART Analyzer, BTS Engineering) and MATLAB software (MATLAB 7.4.0, MathWorks, Natick, MA, USA). Heel strike and toe-off events were determined as in a previous study (32).

For each participant, we rejected the first walking trial and considered the subsequent five trials. To ensure that the gait parameters were captured during steady state walking, the first (acceleration phase) and last (deceleration phase) two steps of each trial were excluded from the analysis. Hence, we considered for analysis only the central two steps of the affected and unaffected sides for each trial, corresponding to approximately 20 steps (on average) for each subject during each session.

Assuming that gait speed was slower in cases in comparison to that of controls and in order to avoid potential velocity bias, the gait speed was matched between groups as follows: for each control group subject, we considered only those trials in which gait speed was within the range identified by the mean gait speed ± S.D. of case group subjects in both baseline and 10-weeks evaluations (33, 34).

#### Spatio–Temporal Parameters

The following temporal parameters were calculated for each subject and session: stance duration (time between two consecutive foot strikes of the same lower limb) expressed as a percentage of the stride duration. The following spatial parameters of gait were computed for each session: step length (m) and step width (m). Furthermore, walking speed (m/s), and cadence (step/min) were calculated for each subject and session.

#### Kinematic Parameters

Euler angles were used to define the 3D relative angular motion of joints of the lower extremities. Kinematic assessment was performed based on the RoM, i.e., the difference between the maximum and minimum angles of the hip, knee, and ankle joints and the trunk. The flexion-extension RoMs of the affected and unaffected hips and of the trunk were computed in the sagittal plane; the trunk RoM was also calculated in the frontal and transverse planes (35). Spatial symmetry assessment was executed through the following asymmetry index (36, 37):

(1) $$\begin{array}{r} \begin{array}{c} spatial-asymmetry=\left(1-\frac{\min\left(AstepLength,\;NAstepLength\right)}{\max\left(AstepLength,\;NAstepLength\right)}\right)\cdot 100 \end{array} \end{array}$$

where AstepLength was the step length of the affected/most affected side and NAstepLength was the unaffected/least affected side.

### Statistical Analyses

*A priori* power analysis using the G^*^Power computer program (38) indicated that a total sample of 34 participants would be needed to detect medium effects (*d* = 0.5) with 80% power using a paired *t*-test between means with α = 0.05.

Changes in gait variables and UPDRS scores at the 10-weeks evaluation were expressed as delta values according to the following formula:

(2) $$\begin{array}{r} \Delta =100\cdot \frac{value_{10week}-value_{baseline}}{value_{baseline}} \end{array}$$

Using this formula, each gait parameter showed improvement in cases if Δ values (either positive or negative), depending on the considered gait variable, were closer to control values. Otherwise, cases were considered to have remained unchanged or worsened. The Shapiro-Wilk test for normal distribution was preliminarily executed. The Student's *t*-test or Mann-Whitney *U* test were used to compare gait parameters between cases and controls. The paired *t*-test or Wilcoxon test were used to detect significant differences between clinical parameters and gait parameters at the baseline and at the 10-weeks evaluation. Cohen's *d* was employed as a measure of effect size to assess small (*d* = 0.2), medium (*d* = 0.5), and large (*d* = 0.8) effects.

An anchor–based method was used to assess the responsiveness of the normalized parameters. Subjects of case group were categorized as improved if their parameters improved and reached the values of parameters in healthy controls at 10-weeks evaluation. Receiver operating characteristic (ROC) curves were plotted and the area under the curve (AUC) of Δ values was calculated to assess responsive ability. A cut–off analysis was performed to identify the minimal clinical important differences (MCIDs) that best differentiated between patients who normalized and patients who did not normalize their values. The optimal MCID evaluation was based on the smallest sum of squares of 1-sensitivity (Se) and 1-specificity (39). Likelihood ratios (LRs) were also calculated and post–test probabilities were inferred by transforming LRs into odds ratios (ORs) through a Fagan's nomogram (40).

Side-dependent gait measures (e.g., step length and hip joint RoM) were separately analyzed and compared between the affected/ most affected side (A) and the unaffected/ least affected side (NA). The chi-square test for categorical variables was used to compare the number of cases which improved and the number of cases which either remained unchanged or worsened. Pearson or Spearman bivariate correlation tests were used to evaluate the correlation between clinical parameters at baseline and Δ values of gait variables. Multiple linear regression analysis with backward selection was performed to evaluate the predictive value of each variable at baseline on improvement of gait variables during the final evaluation.

To validate the multiple regression analysis, we ascertained the independence of observations (i.e., independence of residuals), the linear relationship between the dependent variable and each independent variable and the same between the dependent variables and the independent variables collectively, the homoscedasticity, the absence of multicollinearity and significant outliers, and the normal distribution of residuals. Significance was set at *p* < 0.05 for two-sided tests; the analyses were performed using SPSS 20.0 (IBM) and MedCalc Statistical Software version 18.11.6.

## Results

### Clinical Findings

Fifty patients satisfied the inclusion criteria, and of these, 14 were excluded from the study because 2 of them could no longer be contacted, 4 patients underwent drug modification, 3 patients failed to complete T1 due to transportation problems, 4 patients had medical complications, and 1 patient refused to continue the rehabilitation program. A total of 36 patients (72%) (Figure 1) completed the 10-weeks evaluation (age: 68.83 ± 9.85 years; sex: 16 F, 20 M; weight: 71.68 ± 12.53 Kg; height: 1.64 ± 0.07 m; disease duration: 7.56 ± 4.15 years; levodopa equivalent dose: 571.92 ± 317.2 mg; H-Y stage: 2.26 ± 1.02; UPDRS-II at baseline: 12.61 ± 7.19; UPDRS-II at 10-weeks evaluation: 13.49 ± 6.82; UPDRS-III at baseline: 15.78 ± 6.89; UPDRS-III at 10-weeks evaluation: 13.74 ± 6.03).

The patients were on oral levodopa (18 patients), dopamine agonists (5 patients), or both (13 patients) and were recorded to be in the “ON phase.”

Twenty-two healthy subjects (10 F, 12 M; mean age: 65.6 years; range: 57–75 years; height: 1.67 m; weight: 72.8 kg) were included in the study as a control group.

No significant differences in age, sex, height, or weight (*p* > 0.05) were found between cases and controls. The pharmacological treatment of each case and their H-Y stage remained unchanged at the 10-weeks evaluation when compared to the baseline evaluation. We found a significant decline in the UPDRS-III score (2.04 points) at 10 weeks (*t* = 3.701, *p* < 0.001, Cohen's *d* = 0.315). No significant differences were found in UPDRS-II scores between the baseline and 10-weeks evaluation (*p* > 0.05).

#### Gait Analysis Findings

##### Patients vs. controls

At the baseline evaluation, people with Parkinson's disease showed slower gait speed than controls (0.77 m/s vs. 0.99 m/s, *p* < 0.05), and at matched speed, they showed shorter step length on both sides, reduced hip, knee, and ankle joint RoMs on both sides, reduced trunk rotation RoM, higher cadence, and higher spatial asymmetry index values, almost all of which had large effect sizes (*d* > 0.8; Table 1).

**Table 1:** Comparisons between PD patients and healthy controls both at baseline and 10-weeks follow-up evaluations.

**Parameters**	**Baseline**					**10-weeks follow-up**				
	**Patients**	**Controls**	***t***	***p***	**Cohen's d**	**Patients**	**Controls**	***t***	***p***	**Cohen's d**
Cadence (*n*° step/min)	102.307 ± 16.146	88.280 ± 12.406	3.490	**0.001**	0.970	107.143 ± 15.437	95.573 ± 11.433	2.986	**0.004**	0.851
Speed (m/s)	0.777 ± 0.305	0.786 ± 0.179	−0.124	0.902	0.035	0.864 ± 0.264	0.870 ± 0.169	−0.100	0.921	0.027
R stance duration (%)	63.513 ± 3.954	63.886 ± 2.149	−0.407	0.686	0.117	62.684 ± 3.801	62.633 ± 2.016	0.056	0.955	0.017
L stance duration (%)	63.377 ± 3.687	63.577 ± 2.055	−0.233	0.817	0.067	62.233 ± 3.793	62.410 ± 1.656	−0.202	0.841	0.060
R doub. supp. duration (%)	13.386 ± 3.610	13.982 ± 1.803	−0.719	0.475	0.209	12.342 ± 3.653	12.771 ± 1.804	−0.503	0.617	0.149
L doub. supp. duration (%)	13.344 ± 3.753	13.514 ± 2.471	−0.188	0.852	0.054	13.042 ± 5.249	12.333 ± 2.085	0.590	0.558	0.177
R step length (m)	0.411 ± 0.125	0.487 ± 0.062	−2.651	**0.010**	0.770	0.447 ± 0.121	0.518 ± 0.060	−2.492	**0.016**	0.743
L step length (m)	0.413 ± 0.121	0.493 ± 0.056	−2.921	**0.005**	0.849	0.459 ± 0.110	0.525 ± 0.060	−2.520	**0.015**	0.745
Step width (m)	0.164 ± 0.019	0.156 ± 0.024	1.450	0.153	0.369	0.168 ± 0.021	0.159 ± 0.023	1.570	0.122	0.408
Spatial asymmetry (%)	12.526 ± 11.824	6.764 ± 5.723	2.133	**0.037**	0.620	7.229 ± 6.518	5.496 ± 4.719	1.065	0.291	0.305
R hip flex–ext RoM (°)	32.988 ± 8.449	38.975 ± 3.726	−3.134	**0.003**	0.917	36.001 ± 8.792	40.774 ± 4.120	−2.336	**0.023**	0.695
L hip flex–ext RoM (°)	33.039 ± 9.131	39.923 ± 3.953	−3.341	**0.001**	0.978	35.901 ± 8.911	42.414 ± 5.043	−3.068	**0.003**	3.055
R knee flex–ext RoM (°)	45.202 ± 2.074	52.355 ± 1.597	−13.844	** <0.001**	3.065	45.439 ± 2.647	54.686 ± 1.443	−14.744	** <0.001**	5.276
L knee flex–ext RoM (°)	48.567 ± 1.915	54.075 ± 1.171	−12.148	** <0.001**	3.470	49.876 ± 2.722	56.963 ± 1.061	−11.404	** <0.001**	3.431
R ankle flex–ext RoM (°)	23.730 ± 1.279	26.849 ± 1.772	−7.769	** <0.001**	2.018	23.992 ± 0.948	26.977 ± 1.169	−10.516	** <0.001**	2.805
L ankle flex–ext RoM (°)	24.267 ± 1.064	26.833 ± 1.350	−8.040	** <0.001**	2.111	25.408 ± 0.901	29.157 ± 1.269	−13.555	** <0.001**	3.407
Trunk flex-ext RoM (°)	3.362 ± 1.365	3.468 ± 0.945	−0.320	0.750	0.090	3.948 ± 2.597	4.062 ± 1.848	−0.177	0.860	0.051
Trunk bend RoM (°)	4.008 ± 1.857	4.037 ± 1.589	−0.061	0.951	0.017	4.321 ± 2.355	4.671 ± 2.532	−0.525	0.601	0.143
Trunk rot RoM (°)	6.996 ± 3.169	10.241 ± 3.758	−3.525	**0.001**	0.934	10.033 ± 5.822	12.098 ± 4.586	−1.392	0.170	0.394

Independent sample t-test was used to compare PD patients and healthy controls both at baseline and 10-weeks follow-up evaluations.

Bold p <0.005 are considered statistically significant.

Data are expressed as mean ± SD.

*L, left side; R, right side; RoM, range of motion; doub. supp., double support; bend, bending; flex-ext, flexion-extension; rot, rotation*.

At the 10-weeks evaluation, people with Parkinson's disease showed slower gait speed than controls (0.89 m/s vs. 0.99, *p* < 0.05), and at matched speed, they showed shorter step length on both sides, reduced hip, knee and ankle joint RoMs on both sides, and higher cadence values, all of which had medium or large effect sizes (*d* > 0.6; Table 1). Conversely, no additional significant differences were observed for spatial asymmetry and trunk rotation (Table 1).

**Figure 2:**
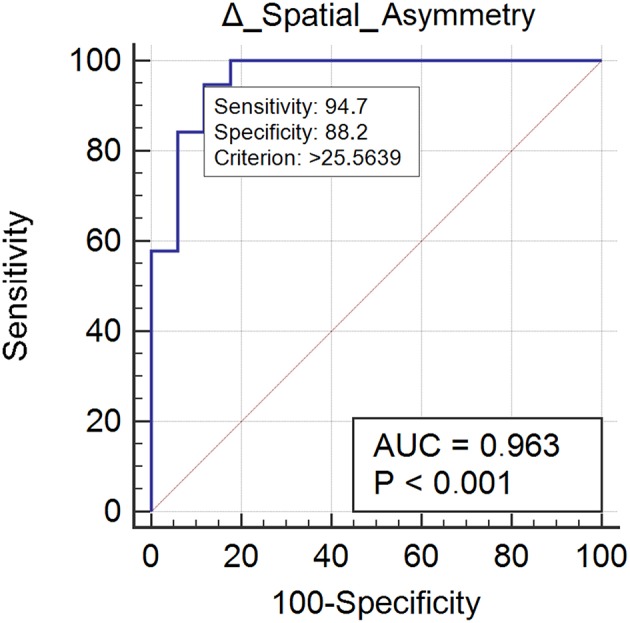
This figure illustrates the Area Under the Receiver Operating Characteristics curve (AUC) of spatial asymmetry improvements. The diagonal purple line represents the non–significance threshold of AUC = 0.50, the blue line represents the true positive rate and the false positive rate at each threshold. Sensitivity and specificity for the optimal MCID (criterion) are reported.

Nineteen subjects (52%) normalized their spatial asymmetry at 10-weeks evaluation. Spatial asymmetry revealed excellent responsive ability (AUC = 0.96) (Figure 2). A spatial asymmetry reduction ≥ 25.56% was found to be the optimal MCID. For a reduction in spatial asymmetry ≥ 25.56, we can rely on 90% in the normalization of spatial asymmetry (Table 2).

**Table 2:** ROC curve and Minimal Clinically Inportant Differences (MCID) analysis of normalized parameters.

**Gait parameter**	**AUC (95% CI)**	**MCID**	**% Se (95% CI)**	**% Sp (95% CI)**	**LR+ (95% CI)**	**LR− (95% CI)**	**PV+ (95% CI)**	**PV− (95% CI)**	**PPR (OR)**	**NPR (OR)**
Δ Spatial asimmetry	0.963 (0.840–0.998)	>25.56	94.74 (74.0–99.9)	88.24 (63.6–98.5)	8.05 (2.2–29.7)	0.06 (0.009–0.4)	90 (70.9–97.1)	93.7 (68.8–99.0)	90% (9.0)	73% (2.7)
Δ Trunk rotation	0.784 (0.616–0.903)	>9.00	95 (75.1–99.9)	56.25 (29.9–80.2)	2.17 (1.2–3.8)	0.089 (0.01–0.6)	73.1 (60.7–82.7)	90 (55.9–98.5)	6% (0.1)	9% (0.1)

*Δ is equal to the difference between the value of each parameter after and before the rehabilitative treatment. The Area Under the ROC Curve (AUC), sensitivity (Se), specificity (Sp), positive (LR+), and negative Likelihood Ratio (LR−), positive (PV+) and negative Predictive Value (PV−), Positive Posterior Probability (PPR), and negative Posterior Probability (NPR) with corresponding Odds Ratios (OR) are reported*.

**Figure 3:**
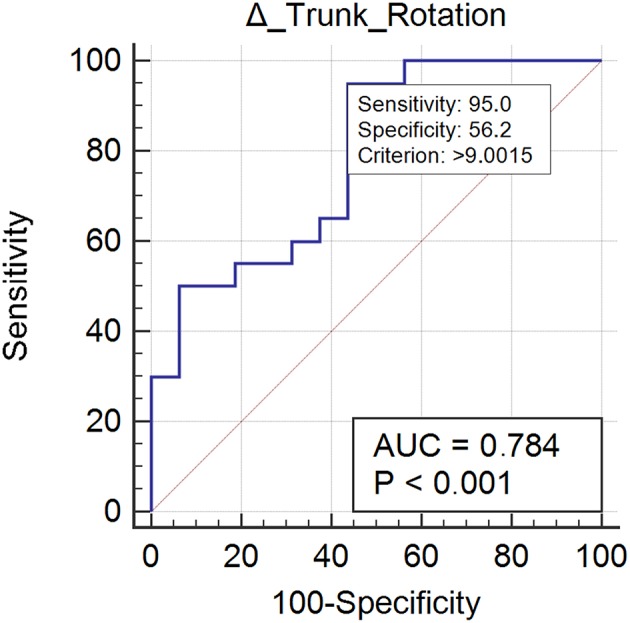
This figure illustrates the Area Under the Receiver Operating Characteristics curve (AUC) of trunk rotation improvements. The diagonal purple line represents the non–significance threshold of AUC = 0.50, the blue line represents the true positive rate and the false positive rate at each threshold. Sensitivity and specificity for the optimal MCID (criterion) are reported.

Twenty case group subjects (55%) reached the control group trunk rotation values. Trunk rotation revealed optimal responsiveness to rehabilitation treatment (AUC = 0.78) (Figure 3). Improvement ≥ 9.00% was found to be the optimal MCID. For an increment ≥ 9.00% in trunk rotation we could be the 73% confidents of normalization of the parameter (Table 2).

##### Ten-week evaluation vs. baseline

**Figure 4:**
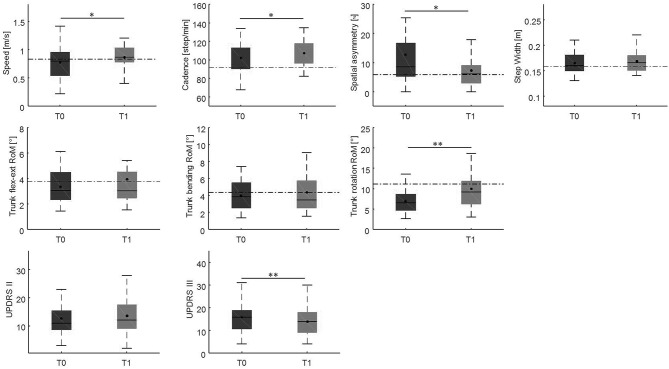
This figure shows the time-distance parameters, spatial symmetry index, and trunk kinematics at baseline and at the 10-week follow-up. For each box, the central black horizontal line indicates the median, the central black dot denotes the mean, and the bottom and top edges of the box indicate the 25 and 75th percentiles, respectively. Whiskers extend to the most extreme data points not considered outliers. Asterisks denote statistically significant differences (*p < 0.05; **p < 0.01). Black horizontal dashed lines represent the mean of each parameter in the healthy control group. flex-ext, flexion-extension; RoM, Range of Motion; T0, baseline; T1, 10-weeks follow-up.

**Figure 5:**
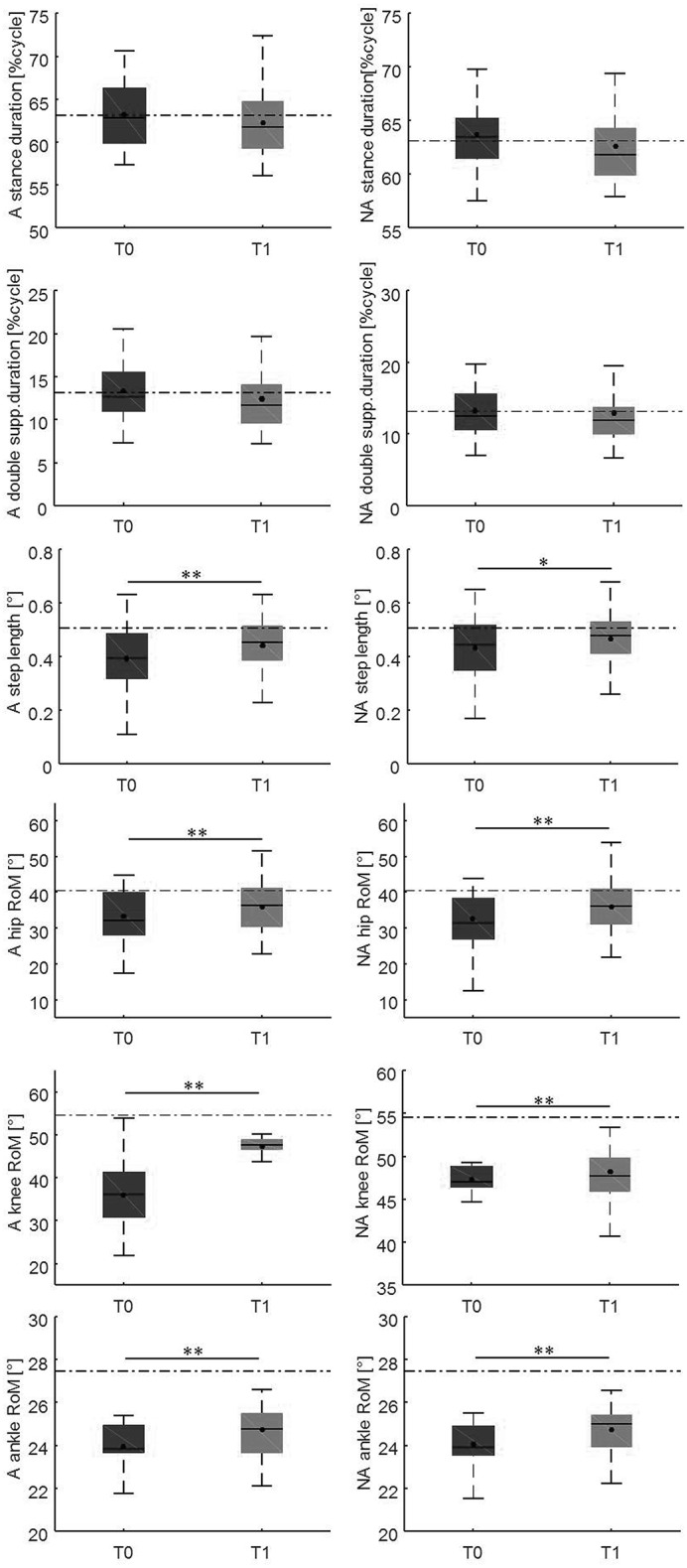
This figure illustrates the side-dependent time-distance parameters and hip, knee, and ankle joint kinematics at baseline and at the 10-weeks follow-up. For each box, the central black horizontal line indicates the median, the central black dot denotes the mean, and the bottom and top edges of the box indicate the 25 and 75th percentiles, respectively. Whiskers extend to the most extreme data points not considered outliers. Asterisks denote statistically significant differences (*p < 0.05; **p < 0.01). Black horizontal dashed lines represent the mean of each parameter in the healthy control group. A, most affected/affected side; flex-ext, flexion-extension; NA, less affected/unaffected side; RoM, Range of Motion; T0, baseline; T1, 10-weeks follow-up.

Changes in gait variables between the baseline evaluation and the 10-weeks evaluation are reported in Figures 4, 5. We found significantly lower spatial asymmetry index values (*t* = −2.614, *p* = 0.013, Cohen's *d* = 0.555), higher cadence values (*t* = −2.180, *p* = 0.036, Cohen's *d* = 0.306), higher speed (*t* = −2.129, *p* < 0.040, Cohen's *d* = 0.305), longer step length in both the affected/most affected (*t* = −3.309, *p* = 0.002, Cohen's *d* = 0.422) and the unaffected/least affected (*t* = −2.271, *p* = 0.029, Cohen's *d* = 0.280) sides, increased hip joint RoM, in both the affected/most affected (*t* = −2.848, *p* = 0.007, Cohen's *d* = 0.306) and the unaffected/least affected (*t* = −3.020, *p* = 0.005, Cohen's *d* = 0.361) sides, increased knee joint RoM in both the affected/most affected (*z* = −4.870, *p* < 0.001, Cohen's *d* = 1.716) and the unaffected/least affected (*z* = −3.158, *p* = 0.002, Cohen's *d* = 0.256) sides, increased ankle joint RoM in both the affected/most affected (*z* = −4.226, *p* < 0.001, Cohen's *d* = 0.667) and the unaffected/least affected (*z* = −3.629, *p* = <0.001, Cohen's *d* = 0.516) sides, and increased trunk rotation RoM (*z* = −2.985, *p* = 0.003, Cohen's *d* = 0.648).

For each gait parameter, we found a significantly greater number of case group subjects who improved (all >64%) compared to those who did not improve, using a chi-square test (Supplementary Material for review).

##### Correlation findings

No significant correlation between the Δ values of the gait variables and the clinical parameters (all *p* > 0.05) were found.

##### Multiple regression findings

According to our multiple linear regression models, some gait parameters were predicted by a set of clinical characteristics which included sex, UPDRS-III or II, disease duration, and H-Y score, as well as each corresponding baseline gait parameter. Specifically, sex combined with UPDRS-III predicted both ΔSpeed and ΔStep length of both sides, while sex combined with UPDRS-II predicted ΔCadence, and age combined with H-Y score and disease duration predicted Δtrunk rotation RoM (Table 3).

**Table 3:** Multiple linear regression analysis for gait parameters improvements.

**Variables**	**B**	**SE**	***p***
**ΔSPEED (m/s)**			
Constant	1.264	0.186	<0.001
UPDRS III	−0.018	0.004	<0.001
Gender (F/M)	−0.248	0.059	<0.001
Speed at baseline (m/s)	−0.446	0.109	0.001
Adjusted *R*^2^ = 0.561, *F* = 8.672, *p* < 0.001			
**ΔCADENCE (step/min)**			
Constant	69.212	14.638	<0.001
UPDRS II	−0.523	0.259	0.056
Gender (F/M)	−8.518	3.534	0.025
Cadence at baseline (step/min)	−0.402	0.114	0.002
Adjusted *R*^2^ = 0.347, *F* = 5.430, *p* = 0.006			
**ΔSTEP LENGTH (MOST AFFECTED SIDE) (m)**			
Constant	0.409	0.059	<0.001
UPDRS III	−0.006	0.001	<0.001
Gender (F/M)	−0.056	0.018	0.004
Step length at baseline (most affected side) (m)	−0.413	0.084	<0.001
Adjusted *R*^2^ = 0.505, *F* = 10.529, *p* = 0.001			
**ΔSTEP LENGTH (LESS OR NOT AFFECTED SIDE) (m)**			
Constant	0.421	0.073	<0.001
UPDRS III	−0.005	0.0002	0.002
Gender (F/M)	−0.077	0.021	0.002
Step length at baseline (less or not affected side) (m)	−0.297	0.089	0.003
Adjusted *R*^2^ = 0.444, *F* = 5.987, *p* = 0.002			
**ΔTRUNK ROTATION ROM (°)**			
Constant	39.059	5.880	<0.001
Hoehn-Yahr stage	−2.170	0.605	0.001
Disease duration (years)	0.392	0.156	0.019
Age (years)	−0.387	0.073	<0.001
Trunk rotation RoM at baseline (°)	−0.955	0.258	0.001
Adjusted *R*^2^ = 0.568, *F* = 10.523, *p* < 0.001			

Δ is equal to the difference between the value of each parameter after and before the rehabilitative treatment.

*The gait variables considered as independent variables are all before the rehabilitative treatment. The unstandardized coefficients (B), with their standard errors (SE) and statistical significances (p), are reported for each model, together with each model's goodness of fitting parameter (adjusted R^2^) and the ANOVA results (F and p)*.

## Discussion

The main findings of this study showed that (1) after a 10-weeks rehabilitation program, almost all gait parameters improved; (2) spatial asymmetry and trunk rotation normalized after rehabilitation; and (3) a set of clinical variables including sex, UPDRS-III and UPDRS-II scores, age, H-Y score, and disease duration can predict the improvement of gait parameters.

At baseline evaluation and unmatched speed, people with Parkinson's disease showed a significantly reduced gait speed compared to controls (0.77 m/s vs. 0.99 m/s). Because most gait parameters are speed-dependent, we matched gait speed for both the evaluations (at baseline and after rehabilitation) in our study. This procedure further improved our understanding of the most relevant impaired gait parameters and those that are most susceptible to improvement by rehabilitation without gait speed as a confound. At matched speed, almost all the gait parameters of the case group were significantly different from those of the control group. Many previous studies did not control for gait speed, resulting in inconsistent results. For instance, the cadence of people with Parkinson's disease was reported to be reduced or normal in gait analysis studies, although it has been long known by clinicians to be increased; e.g., during patients' “festination” (14–16). Our findings confirm that patients with both early and advanced Parkinson's disease show gait impairment (14–18) and that the specific abnormal gait pattern is characterized by reduced gait speed, reduced step length, decreased lower limb joint RoMs, and decreased trunk rotation combined with an increased cadence.

After the 10-weeks rehabilitation program, we found an improvement in all the gait parameters that were impaired at baseline (Figures 4, 5). This finding indicates that the gait parameters that were impaired at baseline are susceptible to improvement by rehabilitation.

Such improvement was observed in a high proportion of patients (>64% for each gait parameter), and interestingly, led to a complete normalization of certain parameters, i.e., spatial asymmetry index and trunk rotation RoM (Table 1). These results suggest that rehabilitation should focus on those gait parameters that are specifically susceptible to normalization and that we should also identify alternative rehabilitative strategies to improve other parameters. We also identified the minimal clinically important differences of improvement in spatial asymmetry and trunk rotation (Table 2). Our study showed that a reduction in spatial asymmetry >25.56% and an improvement in trunk rotation >9.00% over the course of rehabilitation were clinically important in people with Parkinson's disease and could lead to the normalization of these parameters, providing clinicians with thresholds that can guide their interpretation of changes in gait parameters over successive measurements.

Using multiple regression analysis, we found that the delta values of the gait parameters were causally associated with a set of clinical parameters including sex, UPDRS-III or II score, age, H-Y score, and disease duration. These results are in line with those of previous studies reporting female sex, disease stage, and disease duration as negative predictors of physical–functioning in people with Parkinson's disease (41–43). Specifically, sex and H-Y scores seem to be the strongest predictors of gait parameter improvement, accounting for the greatest amount of variation in gait variables that can be explained by the model (Table 3); this suggests that men at earlier disease stages are more prone to improving their gait function after rehabilitation. Bowman et al. (44) reported male sex, higher stages of disease, and more severe mobility limitation as predictors of changes in mobility as assessed by a patient-reported outcome measure, suggesting that results might be different when considering performance mobility tests instead of self–report questionnaires (44). Patients at early stages of disease may report a higher expectation of improvement from rehabilitation treatment than more disabled ones. In this study, we confirmed male sex alone as a positive predictor of gait improvement, and higher disease severity and mobility limitation as negative predictors.

Interestingly, we did not find a bivariate correlation between the clinical variables and gait variables, indicating that no single clinical variable, but rather a set of clinical variables, can predict improvement in gait function.

One of our interesting results is that trunk rotation RoM, which normalizes after rehabilitation, can be predicted by age, H-Y score, and disease duration. Thus, younger patients who are at early stages but have longer disease duration may show greater trunk RoM improvement after rehabilitation. This is an important finding because trunk control is greatly impaired in people with Parkinson's disease (45, 46). It is known that trunk control during gait cycle is functionally important in minimizing the magnitude of linear and angular displacement of the head, ensuring clear vision (47, 48), facilitating the integration of vestibular information (48, 49), contributing to the maintenance of balance (31, 50–52), driving forces for locomotion (53), and creating a more energy-efficient gait pattern (54).

In conclusion, the results from our multiple regression analysis suggest that younger men with Parkinson's disease, who are less severely affected and are at an early disease stage, are more susceptible to improvement in gait function after a short-term (10 weeks) rehabilitation program. Interestingly, the H-Y stage was not the only predictor of gait function improvement. This is noteworthy, because the current European rehabilitation guidelines are mainly planned according to the H-Y staging system.

These findings could allow us to increase our ability to group patients according to their clinical characteristics, with a common aim of focusing the rehabilitative programs on those who are more prone to improvement after short-term rehabilitation. At the same time, we must also focus our efforts on identifying new rehabilitative strategies for patients who are less susceptible to improvement after short-term rehabilitation. For instance, in addition to disease stage, rehabilitative treatment should also be decided according to sex, disease severity, and disease duration. Particular attention should be paid to rehabilitation of the trunk when trying to decide the rehabilitative strategies.

Further multi-center studies with larger samples of patients are needed to better associate the motor improvement induced by rehabilitation to specific rehabilitative techniques.

## Data Availability

The raw data supporting the conclusions of this manuscript will be made available by the authors, without undue reservation, to any qualified researcher.

## Ethics Statement

This study was carried out in accordance with the recommendations of Declaration of Helsinki with written informed consent from all subjects. All subjects gave written informed consent in accordance with. The protocol was approved by the ethics committee Sapienza University of Rome, Policlinico Umberto I (protocol number UP 00263_2019). Prior to taking part in the study, all participants provided written consent after a full explanation of the experimental procedure. This study is registered as a clinical trial in Clinical.trials.gov. (NCT03336307).

## Author Contributions

Research project was conceptualized by MS, GCo, and FP, organized by MS, MB, and CdL, and executed by MS, GCh, SC, CC, and GCa. Statistical analysis was designed by MS and SC, executed by SC, GCh, and CC, and reviewed and critiqued by TV. MS wrote the first draft of the manuscript. It was reviewed by SC, MB, and AR, and critiqued by FP and PC.

### Conflict of Interest Statement

The authors declare that the research was conducted in the absence of any commercial or financial relationships that could be construed as a potential conflict of interest.
